# Integrated transcriptomic and genomic analysis improves prediction of complete remission and survival in elderly patients with acute myeloid leukemia

**DOI:** 10.1038/s41408-020-0332-3

**Published:** 2020-06-11

**Authors:** Albin Österroos, My Björklund, Anna Eriksson, Johan Lindberg, Christer Nilsson, Sylvain Mareschal, Mattias Rantalainen, Henrik Grönberg, Sören Lehmann

**Affiliations:** 1 Department of Medical Sciences, Hematology, Uppsala University, Uppsala, Sweden; 2 Department of Medical Epidemiology and Biostatistics (MEB), Karolinska Institute, Stockholm, Sweden; 3 Center for Hematology and Regenerative Medicine, Department of Medicine, Karolinska Institute, Huddinge, Stockholm, Sweden

**Keywords:** Cancer genetics, Acute myeloid leukaemia, Genetics research

## Abstract

Relevant molecular tools for treatment stratification of patients ≥65 years with acute myeloid leukemia (AML) are lacking. We combined clinical data with targeted DNA- and full RNA-sequencing of 182 intensively and palliatively treated patients to predict complete remission (CR) and survival in AML patients ≥65 years. Intensively treated patients with *NPM1* and *IDH2*^R172^ mutations had longer overall survival (OS), whereas mutated *TP53* conferred lower CR rates and shorter OS. *FLT3-ITD* and *TP53* mutations predicted worse OS in palliatively treated patients. Gene expression levels most predictive of CR were combined with somatic mutations for an integrated risk stratification that we externally validated using the beatAML cohort. We defined a high-risk group with a CR rate of 20% in patients with mutated *TP53*, compared to 97% CR in low-risk patients defined by high expression of *ZBTB7A* and *EEPD1* without *TP53* mutations. Patients without these criteria had a CR rate of 54% (intermediate risk). The difference in CR rates translated into significant OS differences that outperformed ELN stratification for OS prediction. The results suggest that an integrated molecular risk stratification can improve prediction of CR and OS and could be used to guide treatment in elderly AML patients.

## Introduction

Acute myeloid leukemia (AML) is characterized by clonal expansion of early myeloid precursors. As for most hematological malignancies, AML incidence increases with age, whereas survival rapidly decreases in older patients^1,2^. Patients above the age of 65, which constitutes more than 50% of all AML cases, differ considerably regarding patient-specific and leukemia-specific features compared to younger AML patients^2^. In elderly AML patients, favorable cytogenetics are less common whereas adverse cytogenetics and myelodysplastic syndrome (MDS)-related changes are more frequent^3^. Elderly AML patients have higher prevalence of comorbidities and worse performance status, both risk factors for poor outcome^4,5^. Elderly patients more frequently present with a secondary AML, either therapy-related (t-AML) after previous cytotoxic treatment or following an antecedent hematological disorder, (AHD–AML), such as myeloproliferative neoplasms (MPN) or MDS^4,6^. Moreover, age-related sensitivity to drug toxicity and interactions with other drugs that elderly often take simultaneously may exacerbate drug toxicity in this group^3^.

The choice whether to treat older AML patients with intensive induction chemotherapy or palliative treatments remains one of the most difficult clinical dilemmas in AML treatment. Despite its importance, prognostic tools to predict CR in elderly are lacking and treatment decisions are more often guided by the physician’s intuition on patient tolerability of a certain treatment in combination with disease characteristics. Contrary to previous beliefs, evidence suggests that older patients can benefit from more intensive treatment and achieve long-standing remissions^7^.

Although complete remission (CR) rates in general are lower and CR duration shorter, a proportion of patients over 65 years become long-term survivors, making identification of these patients crucial^4,7^. Meanwhile, elderly patients unlikely to respond to treatment should not be subjected to an ineffective intensive treatment causing morbidity, poorer quality of life and mortality^8^. With the advent of novel treatment alternatives for elderly patients such as hypomethylating agents and bcl-2 inhibitors, identification of patients that can benefit from conventional intensive induction is increasingly important.

Few published studies have addressed the prognostic impact of molecular markers in elderly AML patients and RNA-sequencing (RNA-seq) based prognostication is lacking^9–12^. We aimed at combining somatic mutations, RNA-seq, and clinical data in a nationwide cohort of AML patients ≥65 years to identify features indicative of CR achievement and/or predictive of overall survival (OS) in intensively and palliatively treated patients.

## Patients and methods

### Patients and treatments

Pretreatment bone marrow or peripheral blood samples were collected from 204 consecutive AML patients ≥65 years in Sweden between February 1997 and August 2014. One hundred and eighty-two patients were ultimately included after exclusion of patients with acute promyelocytic leukemia (*n* = 2), incorrect diagnosis (*n* = 1), lack of clinical data (*n* = 1), and lack of molecular data (*n* = 18) (Supplementary Fig. S1). Mononuclear cells were Ficoll separated and stored at −180 °C until use.

Patients received either intensive induction chemotherapy according to national treatment guidelines (induction course with daunorubicin 60 mg/m^2^/day on days 1–3 and cytarabine 1 g/m^2^ twice daily on days 1–5 with 1–2 similar consolidation courses), treatment with hypomethylating agents or palliative care. The latter included treatment with hydroxyurea, low-dose cytosine arabinoside, and/or transfusions only. The decision how to treat the patient was taken at the discretion of the treating physician.

The cohort included unselected consecutive AML patients including AHD–AML and t-AML. Patient records and the Swedish Adult Acute Leukemia Registry were used to retrieve clinical data. Ethical approval was given by the regional ethical review board in Stockholm, Sweden, and the study followed the Declaration of Helsinki including informed consent from all subjects.

### Risk stratification

Stratification of risk was performed as described in Supplementary Methods.

### Analyses of somatic mutations and RNA-sequencing data

Sample preparation, sequencing procedures, and preprocessing have been previously described^13^. Demographic information, somatic mutations and normalized RNA-sequencing counts of included patients are openly accessible (10.5281/zenodo.292986)^14^. Details on bioinformatic processing are described in Supplementary Methods.

### Statistical analyses

Intensively and palliatively treated patients were compared using Fisher’s exact test for categorical and the Mann–Whitney U test for continuous variables. OS was measured from date of diagnosis until death from any cause. The predetermined follow-up time was maximally 5 years. Patients alive at last follow-up were censored. Patients who underwent allogeneic transplantation were censored at the time of transplantation but analysis was also performed without censoring at transplantation.

Definitions for relapse-free survival (RFS) and CR, Cox proportional hazards regression analyses and conditional inference tree (CIT) analyses are described in Supplementary Methods. Kaplan–Meier analyses were used for estimations of OS and log-rank test was used for group comparisons of OS. All statistical analyses were two-sided with *P* < 0.05 considered statistically significant. Our proposed risk stratification was externally validated using the beatAML cohort available online (vizome.org/aml)^15^.

## Results

### Characterization of the cohort

In total, 182 patients aged 65 years or older were included (Supplementary Fig. S1) of whom 50% were women. Patient characteristics are described in Table 1 while laboratory chemistry values at diagnosis are shown in Supplementary Table S1. The median age in the cohort was 74 years (range 65–96 years). AHD–AML constituted 15% of the cases whereas t-AML represented 11%. Approximately one-fifth (23%) of the patients harbored a complex karyotype whereas 48% had a normal karyotype. Among patients treated with intensive chemotherapy, 59% achieved complete remission and the median overall survival was 8.2 months when including all treatment regimens (median follow-up time 57 months).

**Table 1:** Clinical and cytogenetic characterization, *n* (%).

Variable	All patients*n* = 182	Intensive treatment*n* = 130	Palliative treatment*n* = 45	*P*-value^a^
Age (median, range)	74 (65–96)	72 (65–85)	81 (65–96)	<0.001
Women	92 (50.5)	66 (50.8)	22 (48.9)	0.86
WHO status				0.001
0	32 (18.6)	30 (24.0)	1 (2.5)	
1	89 (51.7)	65 (52.0)	19 (47.5)	
2	30 (17.4)	17 (13.6)	12 (30.0)	
3	16 (9.3)	11 (8.8)	5 (12.5)	
4	5 (2.9)	2 (1.6)	3 (7.5)	
AML etiology				0.02
De novo	132 (73.3)	102 (78.5)	25 (58.1)	
AHD–AML	28 (15.6)	15 (11.5)	12 (27.9)	
Therapy-related	20 (11.1)	13 (10.0)	6 (14.0)	
ELN risk				0.03
Favorable	29 (17.1)	25 (20.0)	3 (7.7)	
Intermediate	50 (29.4)	41 (32.8)	8 (20.5)	
Adverse	91 (53.5)	59 (47.2)	28 (71.8)	
Normal karyotype	75 (48.4)	62 (53.0)	11 (34.4)	0.01
Complex karyotype^b^	35 (22.6)	23 (19.7)	11 (34.4)	0.38
Acquired mutations (median, range)	3 (0–8)	3 (0–8)	3 (0–6)	0.42
Allogeneic stem cell transplant	8 (4.4)	8 (6.2)	0 (0.0)	0.11
Achieved complete remission	78 (42.9)	77 (59.2)	1 (2.2)	<0.001
Median overall survival (months)	8.2	11.4	1.8	<0.001
% alive at 1 year (95% CI)	36.2 (29.5–44.3)	46.1 (38.0–55.9)	7.6 (2.6–22.0)	
% alive at 3 year (95% CI)	14.4 (9.6–21.4)	19.4 (13.2–28.6)	0	

*WHO* World Health Organization, *AHD–AML* AML with an antecedent hematological disorder, *ELN* European LeukemiaNet, *CI* confidence interval.

^a^*P*-values for comparisons between treatment groups using Fisher’s exact test for categorical variables, the Mann–Whitney U test for continuous variables and the log-rank test for overall survival comparison.

^b^Complex if three or more changes; No available data on WHO status for 10 pts, AML etiology for 2 pts, ELN category for 12 pts, karyotype for 27 pts.

**Fig. 1:**
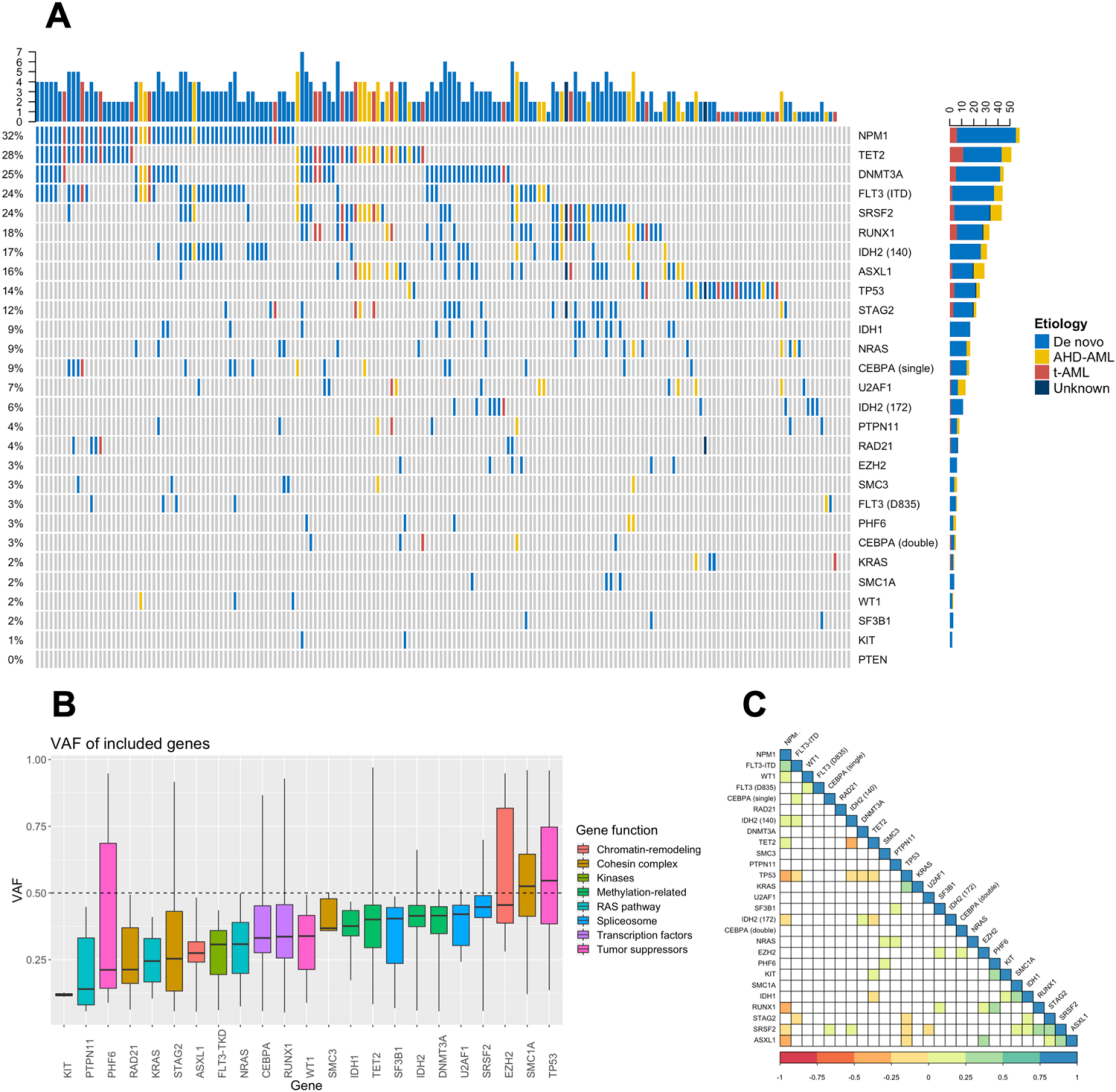
Mutations in elderly AML patients. **a** Oncoplot for all patients included in the study (*n* = 182). Each column represents one patient and each row corresponds to the presence of somatic mutations in defined genes. Colors represent AML etiology. **b** Boxplots showing variant allele fractions (VAFs) of included genes. **c** Correlation plot of statistically significant correlations between gene mutations. AHD–AML AML with an antecedent hematological disorder, t-AML therapy-related AML, ITD internal tandem duplication, TKD tyrosine kinase domain.

The median number of somatic mutations per patient was 3 (range 0–8). The most commonly mutated genes in the whole cohort were *NPM1* (32%), *TET2* (28%), and *DNMT3A* (25%) (Fig. 1a, Supplementary Table S2), while the most common mutations in patients with de novo AML were *NPM1* (37%), *DNMT3A* (28%), and *FLT3-ITD* (26%). The median variant allele fraction (VAF) for all included genes was 0.35 (*FLT3-ITD* and *NPM1* mutations were excluded from the VAF analyses due to their nature as indels). Three of the five genes with lowest median VAFs were involved in signaling kinases (*KIT*) or the RAS pathway (*PTPN11* and *KRAS*) (Fig. 1b) while the highest VAFs were seen for *TP53*, *SMC1A*, *EZH2*, and *SRSF2*.

Pairwise significant positive correlations were seen for 32 gene mutation pairs whereas significant negative correlations were observed for 16 pairs (Fig. 1c). Mutated *NPM1* correlated positively with mutations in *FLT3-ITD*, *WT1*, *IDH2*^140^, and *TET2*. On the contrary, mutated *NPM1* was negatively correlated with mutations in *TP53*, *IDH2*^R172^, *RUNX1*, *SRSF2*, and *ASXL1*. *TP53* mutations displayed the highest number of negative correlations, including mutations in DNA methylation regulating genes (*DNMT3A*, *TET2*, and *IDH2*^140^), but also with mutated *STAG2*, *SRSF2*, *ASXL1*, and *FLT3-ITD*. The only positive correlation with mutated *TP53* was with mutated *KRAS*.

### Patient characteristics and outcome

Frontline therapy consisted of intensive chemotherapy in 130 (71%) patients, palliative treatment in 45 (25%), and hypomethylating agents (HMA) in 7 (3.9%). Comparisons between intensively and palliatively treated patients are included in Table 1, Supplementary Table S1 and Table S2. Only patients with palliative and intensive treatment are further described related to CR and OS due to the low number of HMA-treated patients since HMAs were not widely used in Sweden during the inclusion period^16^.

Palliatively treated patients were older compared to intensively treated patients (median 81 years vs. 72 years, respectively; *P* < 0.001). Palliated patients had higher bone marrow blast counts at time of diagnosis (median 70% vs 54%; *P* 0.03), worse WHO performance status (50% vs 24% for WHO status 2–4; *P* < 0.001), lower prevalence of normal cytogenetics (34% vs 53%; *P* 0.01), higher frequency of AHD–AML (28% vs 12%; *P* 0.02), and more adverse ELN (72% vs 47%; *P* 0.01) compared to intensively treated patients. There was no difference in the number of mutations between intensively and palliatively treated patients (Table 1, Supplementary Table S2, Fig. S2a, b).

Only one patient (2.2%) achieved CR among palliatively treated patients, in contrast to 77 patients (59%) among intensively treated patients. Median OS was 1.8 months for palliatively treated patients and 11.4 months for intensively treated. Intensive treatment conferred longer OS compared to palliative treatment in all mutational subgroups including patients with mutated *TP53*, while OS in mutated *TP53* was not significantly better compared to palliatively treated patients with wild-type *TP53* (log-rank *P*-value 0.15) (Supplementary Fig. S3).

### NPM1, IDH2^R172^, and TP53 mutations are predictive of survival in elderly AML patients

Clinical factors significantly predictive for poor OS in univariate Cox analysis in intensively treated patients were male gender, high lactate dehydrogenase, poor WHO performance status, and high ELN risk, while low hemoglobin and AHD–AML showed borderline significance (*P*-values 0.08 and 0.06, respectively) (Supplementary Table S3).

Among mutations in univariate analysis, *NPM1* mutations conferred better OS (hazard ratio (HR) 0.50 (95% confidence interval (CI) 0.32–0.79)), *TP53* mutations worse OS (HR 1.73 (95% CI 0.98–3.04)) while *IDH2*^R172^ mutations showed borderline significance for better OS (HR 0.50 (95% CI 0.22–1.14)) and *ASXL1* for worse OS (HR 1.73 (95% CI 0.98–3.04) (Supplementary Table S4).

Clinical and mutational data were then included in an age-adjusted multivariable Cox regression analysis for OS in intensively treated patients (Table 2). In the multivariate analysis, mutated *NPM1* and *IDH2*^R172^ were associated with improved OS (HR 0.15 (95% CI 0.06–0.35) and 0.23 (95% CI 0.06–0.86), respectively), whereas mutated TP53 had a significant negative impact on OS (HR 2.20 (95% CI 1.05–4.61). Among clinical variables, poor WHO performance status and high lactate dehydrogenase remained as poor prognostic factors in the multivariable model with HRs of 1.83 (95% CI 1.19–2.83) and 1.07 (95% CI 1.02–1.13), respectively.

**Table 2:** Multivariable Cox regression analyses for overall survival.

Variable	Multivariable Cox regression of OS in intensively treated patients, *n* = 76			Multivariable Cox regression of OS in palliatively treated patients, *n* = 34		
	HR	95% CI	*P*-value	HR	95% CI	*P*-value
Age (continuous)	1.02	0.95–1.08	0.63	0.99	0.92–1.08	0.88
WHO status (continuous)	1.83	1.19–2.83	0.01	1.50	0.91–2.48	0.11
*TP53* (mutated vs wild-type)	2.20	1.05–4.61	0.04	3.97	1.33–11.81	0.01
*NPM1* (mutated vs wild-type)	0.15	0.06–0.35	<0.001	–	–	–
*IDH2*^*R172*^ (mutated vs wild-type)	0.23	0.06–0.86	0.03	–	–	–
*FLT3-ITD* (mutated vs wild-type)	–	–	–	7.62	2.40–24.21	<0.001
Lactate dehydrogenase (continuous, μkat/L)	1.07	1.02–1.13	0.004	–	–	–
Karyotype (normal vs abnormal)	–	–	–	0.19	0.07–0.55	0.002

*OS* overall survival, *CR* complete remission, *CI* confidence interval, *WHO* World Health Organization, *ITD* internal tandem duplication.

Factors significantly associated with RFS in univariate Cox analyses were mutated *NPM1*, which was associated with longer RFS (HR 0.51 (95% CI 0.29–0.91)) and mutated *TP53* that conferred shorter RFS (HR 4.75 (95% CI 1.41–15.99)) (Supplementary Table S5). Early death, defined as death within 30 days from diagnosis, was examined with regard to mutational status (Supplementary Table S6). Sixteen percent of intensively treated patients died within 30 days while no single gene mutation was significantly associated with early death.

### FLT3-ITD and TP53 mutations predict survival in palliatively treated patients

In univariate analysis, the only mutation significantly associated to OS in palliatively treated patients was *FLT3-ITD* with a median OS of 0.7 months compared to 2.5 months in cases with wild-type *FLT3* (HR of 3.88 (95% CI 1.88–8.02)) (Supplementary Fig. S4 and Table S7). Clinical factors with a poor prognostic impact in palliatively treated patients were high lactate dehydrogenase and abnormal karyotype (Supplementary Table S8).

Using age-adjusted multivariate Cox regression analysis, *FLT3-ITD* (HR 7.62 (95% CI 2.40–24.21)) and mutated *TP53* (HR 3.97 (95% CI 1.33–11.81)) were significantly associated with shorter OS while a normal karyotype conferred a better prognosis compared to an abnormal karyotype (HR 0.19 (95% CI 0.07–0.55).

### Mutations in NPM1 and TP53 influence CR rates in elderly AML patients

Whether to give intensive treatment or not to elderly AML patients remains one of the most challenging questions in AML care. In this cohort, intensively treated patients who did not achieve CR had a median OS of 1.6 months (or 48 days) compared to 23 months for those who achieved CR (Supplementary Fig. S5) with an HR of 6.70 (95% CI 4.30–10.45) for OS for non-CR patients (Supplementary Table S3).

In univariate analyses, *NPM1* mutations were significantly associated with an increased chance of achieving CR (odds ratio (OR) 0.46 (95% CI 0.20–0.99)) while *TP53* mutations were significantly associated with a lower CR rate (OR 8.00 (2.37–36.71)) (Supplementary Table S9). Male gender, worse WHO performance score and ELN high risk were significantly associated with lower chance of CR (Supplementary Table S10). None of the available clinical laboratory values were significantly associated with CR rate (Supplementary Table S11).

### Differentially expressed genes associated with CR achievement in elderly AML patients

**Fig. 2:**
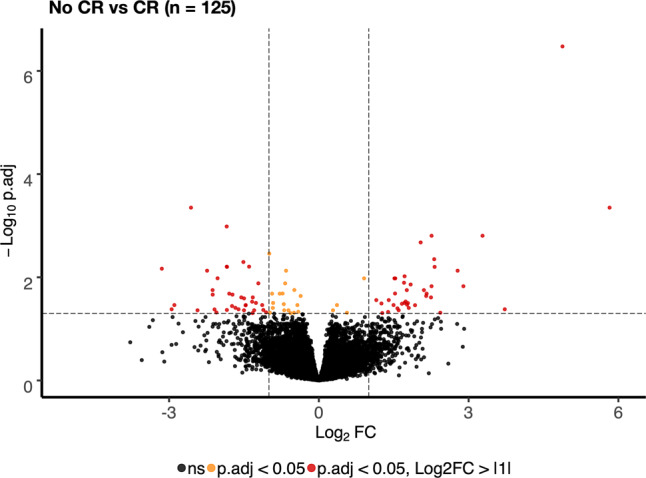
Differentially expressed genes, CR vs. no CR. Volcano plot showing differentially expressed genes in pretreatment samples in intensively treated patients who achieved complete remission (CR; *n* = 48) vs. patients who did not achieve CR (*n* = 77). The −log10 false discovery rate (FDR) adjusted *P*-value (p.adj) is plotted against the log2 fold-change (log2 FC) between the two groups. Ninety-six genes were found to be significantly differentially expressed between groups using <0.05 FDR cutoff, indicated by a dotted horizonal line; the log2 FC > 1 is indicated by the dotted vertical lines. ns not significant, CR complete remission.

To identify more factors that predict the chance of achieving CR in elderly AML patients, we used RNA-sequencing data from intensively treated patients for differential expression analysis. High-quality RNA-sequencing data were available from 125 patients. Ninety-six genes were significantly differentially expressed in pretreatment samples between patients who did achieve CR (*n* = 48) vs. patients who did not achieve CR (*n* = 77), using a false discovery rate (FDR) cutoff of <0.05 (Fig. 2).

The 20 most significant differentially expressed genes (DEGs) are listed in Table 3, along with previously described associations with solid and hematological malignancies. The majority of the genes have been implicated in malignancies and some of them have previously been associated with clinical outcome, such as *BCL2L10*^17,18^, *ZBTB7A*^19^, and *BAG3*^20^. However, several genes with no previous link to clinical outcome in malignancies, including AML, were identified (Table 3, Supplementary Table S12).

**Table 3:** The 20 most significant genes from RNA-seq analysis.

Gene	Log_2_ fold-change (no CR vs CR)	Adjusted *P*-value^a^	Previous association with solid malignancy^b^	Previous association with hematological malignancy^b^
*ADAMTS2*	2.32	0.006	+	−
*AVPR1A*	2.78	0.007	+	−
*BAG3*	−1.85	0.006	+	+
*BCL2L10*	2.26	0.002	+	+
*DNAH2*	4.88	<0.001	−	+
*EEF1A2*	−1.84	0.006	+	−
*EEPD1*	−0.99	0.003	−	−
*FCER1A*	1.72	0.01	−	−
*FOXF2*	5.82	<0.001	+	−
*HSPA6*	−2.24	0.007	+	−
*ITLN1*	−2.56	<0.001	+	−
*MET*	−2.03	0.01	+	+
*MIR3135A*	−1.51	0.005	−	−
*MYRIP*	2.04	0.002	+	−
*NKAIN2*	3.28	0.002	+	−
*PLIN2*	−1.40	0.006	+	+
*RP11–883A18:3*	−3.15	0.007	−	−
*SIGLEC1*	2.31	0.004	+	−
*TNFSF14*	−1.85	0.001	+	−
*ZBTB7A*	−0.66	0.007	+	+

*CR* complete remission.

^a^Adjusted by the Benjamini–Hochberg procedure.

^b^Literature search as defined in Supplementary Methods with additional information including references found in Supplementary Table S12.

### Integrated transcriptomic and genomic sequencing for CR prediction

**Fig. 3:**
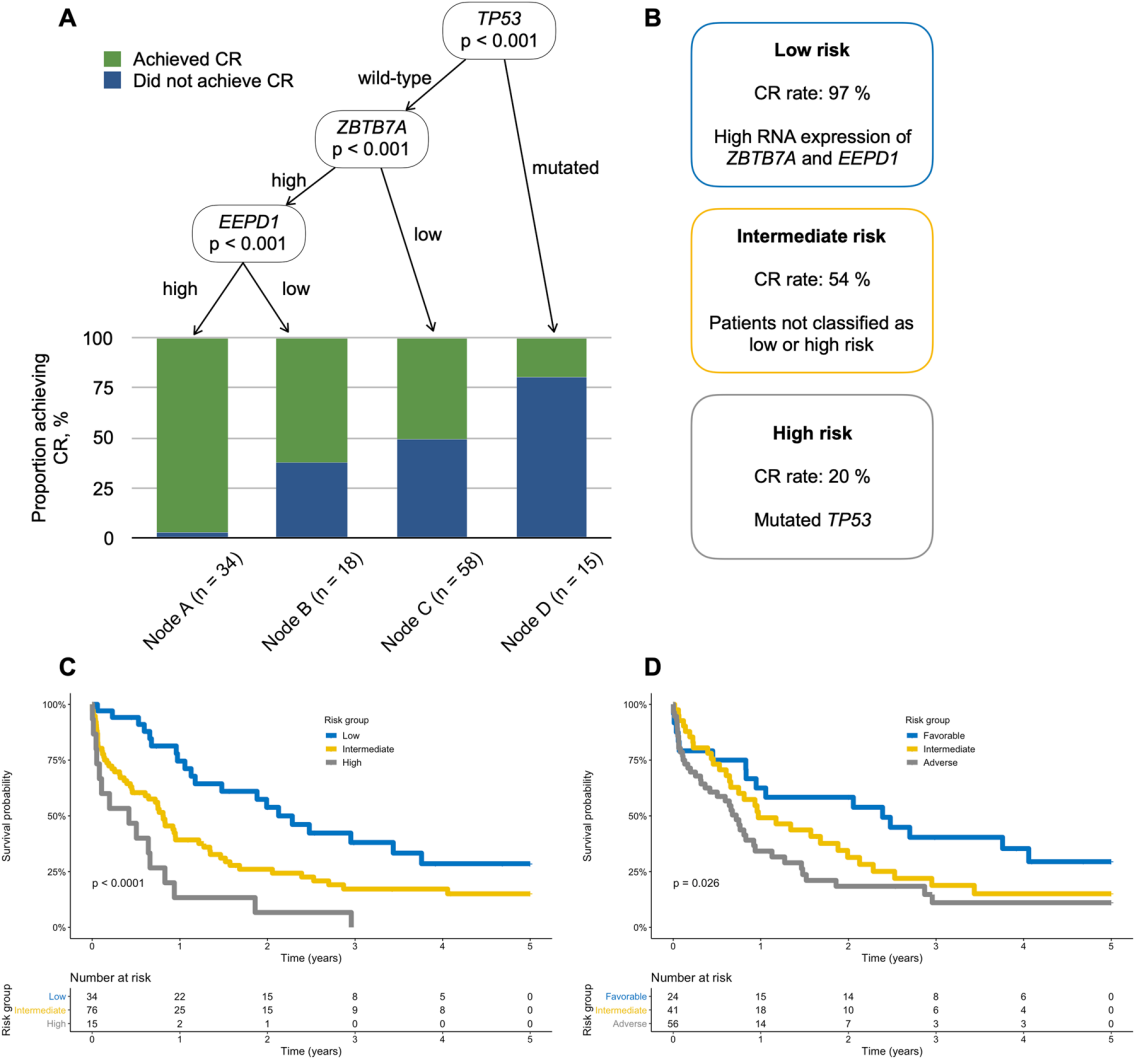
Integrated risk stratification of elderly AML patients. **a** Conditional inference tree diagram for intensively treated patients with complete remission (CR) as outcome. Green color represents proportion of patients achieving CR, blue color represents proportion of no CR. “High” and “low” expression represents expression values above and below median, respectively, based on RNA-sequencing data. **b** Definition of and CR rates in low-, intermediate- and high-risk patients in the proposed risk stratification model. **c** Overall survival for the risk groups in the integrated risk stratification. **d** Overall survival for the same patients based on stratification according to ELN risk. mut mutated, wt wild-type.

Conditional inference trees were employed to find an optimal risk-stratifying algorithm for the chance of achieving CR in intensively treated patients. Somatic gene mutations and gene expression by RNA-seq were included based on the obtained associations with CR in univariate and multivariate analyses. The final decision tree algorithm is depicted in Fig. 3a.

When integrating DEGs with somatic mutations to define gene expression profiles with the strongest predictive power for CR, two genes were shown to add the most independent value to the algorithm. High expression (>median) of zinc finger and BTB domain containing 7A (*ZBTB7A*) together with high expression of endonuclease/exonuclease/phosphatase family domain containing 1 (*EEPD1*) (with no *TP53* and regardless of any other somatic mutation) defined the good-risk group with the highest chance of CR. Patients in the poor-risk group were defined only by mutated *TP53* while the intermediate-risk group was constituted by patients not fulfilling the criteria for good- or poor risk (Fig. 3b).

The three risk groups were defined as good-, intermediate-, or poor risk, with CR rates of 97%, 54%, and 20%, respectively, (constituting 27%, 61%, and 12%, respectively, of the intensively treated patients) with significantly lower chances of reaching CR in high and intermediate-risk groups (RR 0.22 (95% CI 0.07–0.45). The obtained risk groups for CR achievement also translated into differences in OS (Fig. 3c). Median OS estimates were 26, 9.9, and 5.1 months for the good-, intermediate-, and poor-risk group, respectively. Our stratification showed better discrimination between risk groups for OS compared to the current ELN classification (Fig. 3d) which obtained median OS of 29, 12, and 8.8 months for the favorable-, intermediate-, and adverse-risk groups, respectively. The ELN classification neither significantly discriminated between favorable-, and intermediate-risk (log-rank *P* = 0.14), nor between intermediate- and adverse-risk (log-rank *P* = 0.10) in this elderly cohort.

For validation of the CR score, we used the beatAML cohort^15^, the only currently available independent AML data cohort with RNA-seq data also including elderly patients. Due to the low number of elderly patients also in beatAML, we selected patients ≥50 years with non-APL AML from the beatAML cohort to externally validate our results. Ninety-four intensively treated AML patients ≥50 years with available RNA-seq on diagnostic samples were included. CR rates were 90%, 63%, and 43% for our proposed good-, intermediate-, and poor-risk group, respectively, in the beatAML cohort with significantly lower chances of reaching CR in higher-risk groups (RR 0.54 (95% CI 0.23–0.88) with good-risk group as baseline) (Supplementary Fig. S6). Importantly, the high CR rate in the good-risk group in the beatAML cohort could verify the role of expression profiling for CR prediction. The C statistic for the original predictive stratification was 0.93 compared to 0.81 for the beatAML cohort indicating good discriminative capacity.

## Discussion

AML is a disease of the elderly, with a median age of 72 years at diagnosis^6^. However, outcomes have rarely been characterized based on mutational status in older patients and gene expression data by RNA-seq in this AML population is lacking. Here, we describe the mutational landscape of a well-characterized population-based cohort of 182 consecutive AML patients ≥65 years with both intensively and palliatively treated patients. With the aim to find a better tool for CR prediction in elderly, we propose a risk-stratifying algorithm for CR achievement based on integrated analysis including gene mutations from DNA sequencing and gene expression levels by RNA-seq.

Prognosis was overall poor in the cohort with a median OS of 8.2 months. Intensively treated patients achieving CR showed considerably longer OS (23 months) compared to patients not achieving CR (1.6 months). In the era of hypomethylating agents, the role of achieving CR has been discussed^21^ but the remarkably large difference observed in this study highlights the importance of achieving CR when possible in the setting of intensive chemotherapy treatment.

In line with previous studies focusing on elderly AML patients, the most frequently mutated genes were *NPM1*, *TET2*, *DNMT3A*, and *FLT3-ITD*^9–12^. Of somatic mutations, we show that *NPM1*, *IDH2*^R172^, and *TP53* add prognostic value when predicting OS after intensive chemotherapy, although the previously reported positive impact of *IDH1* mutations^12^ could not be confirmed. The positive impact of *NPM1* and *IDH2*^R172^ and negative impact of *TP53* has previously been reported but not uniformly so by all previous studies on elderly AML patients^9–12^, possibly due to diversity in patient populations, sensitivity of mutational screening and differences in patient numbers.

Studies of predictive factors for outcome in palliatively treated patients are rare and molecular data on this patient group is lacking. Both *TP53* and *FLT3-ITD* mutations predicted poor survival, with the effect of *FLT3-ITD* seemingly more prominent, probably explained by the known association to a proliferative disease, which may be more deleterious without chemotherapy^22,23^. Strikingly, there was no significant difference between intensively treated patients with *TP53* mutations compared to wild-type *TP53* patients with palliative treatment, further underlining the poor outcome in *TP53* mutated patients treated by conventional chemotherapy.

The most important objective in the study was to identify factors associated with the chance of achieving CR with the ultimate aim to improve identification of elderly patients that benefit from intensive induction chemotherapy. To further improve CR prediction, we analyzed RNA-seq data from the same patient cohort and first defined genes associated with CR achievement in intensively treated patients. Relatively few genes (~100) were found to be differentially expressed between CR and non-CR patients. Several of the identified genes have previously been associated to malignancies with previous reporting of prognostic or predictive impact for a number of identified DEGs.

When integrating the transcriptomic and mutational data with the aim to create an algorithm for CR prediction, two genes, *ZBTB7A* and *EEPD1*, added the most value to the molecular stratification. Patients with high expression of both genes but without *TP53* mutations formed a good-risk group with a CR rate of 97%. Proper validation of the integrated stratification was challenging since transcriptomic data on elderly AML patients is scarce. For validation, we used the beatAML cohort as it has transcriptomic as well as CR data and includes older AML patients, although the age limit had to be set to ≥50 years to get enough patient numbers to match our cohort. Importantly, the validation confirmed the power of our stratification model for CR prediction between risk groups, including the separation of the good-risk patients based on transcriptomic data. This confirms the role of *ZBTB7A* and *EEPD1* expression within the stratification. Both these genes have shown to be involved in maintaining genomic integrity which can explain a differential sensitivity to chemotherapy. *EEPD1* functions as a gatekeeper in the homologous recombination (HOR) fork repair pathway^24,25^. *EEPD1* depletion has been shown to tilt the repair of double-strand breaks (DSBs) from HOR to the more error-prone process of non-homologous end-joining (NHEJ)^26,27^.

*ZBTB7A* encodes for leukemia/lymphoma-related factor (LRF), a member of the POZ/BTB and Krüppel family of transcription factors. LRF plays a major role in the repair of DSBs. LRF stabilizes the DNA-dependent protein kinase complex that in turn is pivotal for NHEJ in places of DSBs^28^. Accordingly, low RNA expression of *ZBTB7A* has been associated with more aggressive disease stages in solid tumors including prostate cancer^29^, gastric cancer^30^, and malignant melanoma^31^. In AML, *ZBTB7A* has been shown to act as a transcription factor with antiproliferative effects^19^. It also plays a role in hematopoietic lineage fate decisions^19,32^, especially in the erythroid and lymphoid compartments^33^. Mutations in *ZBTB7A* have been associated with core-binding factor AML with RUNX1-RUNX1T1/t(8;21)(q22;q22)^19^,^34–36^.

The difference in CR rate between the risk groups, using this integrated algorithm, also translated into differences in OS. When comparing the stratification in this study to the ELN stratification, our integrated stratification could identify significant OS differences between groups when ELN failed to do so. This suggests that transcriptomic analysis could improve risk stratification also for survival predictions in elderly AML patients and points to the previously reported limitations of ELN stratification in elderly AML patients^9,12^.

As costs for RNA-seq are decreasing and bioinformatic knowledge and pipelines are being developed^37^, our study shows one of the potential clinical uses of applying RNA-seq for AML patients. Other clinical applications of RNA-seq include a detailed view of genomic and cytogenetic aberrations as well as the identification of distinct molecular signatures with prognostic and/or predictive impact. Transcriptome analyses will likely be part of future upfront AML testing. While we were able to validate and confirm our score in an independent AML cohort, the score needs to be further studied and confirmed in other independent cohorts in elderly AML patients either by applying RNA-seq or real-time polymerase chain reaction (RT-PCR) for targeted genes.

In conclusion, this is the first study integrating mutational and transcriptomic data to predict outcomes in elderly AML patients. In addition to identifying mutations in *TP53*, *IDH2*^R172^, and *NPM1* as the most predictive mutations for clinical outcome in elderly AML, it shows the potential power of including transcriptomic data in the prognostic evaluation. Information on the chance of a patient achieving CR could be of major importance when selecting intensive induction chemotherapy or other treatments in this patient group. This information may also be of importance when new treatments with hypomethylating agents as well as bcl-2 inhibitors are introduced in this patient category.

## Supplementary information

Supplementary

## Data Availability

The Clinseq AML dataset is available in the Clinseq AML repository at Zenodo. The beatAML dataset used for external validation is available at http://vizome.org/aml.

## Code availability

Relevant code is available from the corresponding author on reasonable request.
