# Supplementary material for: Integrated transcriptomic and genomic analysis improves prediction of complete remission and survival in elderly patients with acute myeloid leukemia
Source: Blood Cancer J. 2020 Jun 11;10(6):67. doi: 10.1038/s41408-020-0332-3 (PMC7289793; doi:10.1038/s41408-020-0332-3)
Supplement: Supplementary file 1 — Supplementary [file 41408_2020_332_MOESM1_ESM.pdf]

Supplementary

Integrated transcriptomic and genomic analysis improves complete remission and survival predictions in elderly patients with acute myeloid leukemia

Supplementary Methods	2
Supplementary Tables	4
Supplementary Figures	18
Supplementary References	23

Supplementary Methods

Definition of complete remission and relapse-free survival

Complete remissions required an absolute neutrophil count > 1 x 10^9/L, a platelet count > 100 x 10^9/L, no Auer rods, < 5 % bone marrow blast cells, no leukemic blasts in peripheral blood, no evidence of extramedullary leukemia and no need of erythrocyte transfusions.

Relapse-free survival was measured from date of complete remission until date of relapse or death from any cause.

Risk stratification of patients

Risk status was determined from cytogenetic and molecular aberrations in pretreatment bone marrow samples according to the European LeukemiaNet (ELN) guidelines[1]. Allelic fractions for *FLT3-ITD* were not available as this analysis was not part of clinical routine during the studied time period. Hence, patients with normal karyotype with mutated NPM1 and without *FLT3-ITD* were risk classified as "Favorable". Patients with normal karyotype with other combinations of NPM1 and FLT3-ITD were classified as "Intermediate". Complex karyotype was defined as ≥ 3 cytogenetic aberrations lacking WHO-defined recurring inversions or translocations.

Multivariable analyses

All statistical analyses were performed using the computing environment R version 3.5.1[2]. Prognostic factors for OS were investigated via univariate and multivariable Cox proportional hazards regression analyses in both intensively and palliatively treated patients. Variables with P < 0.2 in univariate analyses were included as covariates for the multivariate analyses followed by backward selection. Laboratory variables could unfortunately not be included for palliative patients due to missing data. The final models were adjusted for age at diagnosis. Only patients with full data on the selected initial variables were included.

Included covariates before backward selection were

- gender, hemoglobin, lactate dehydrogenase, karyotype (normal vs abnormal), WHO status, ELN risk (low/intermediate vs adverse), AML etiology, *ASXL1*, *TP53*, *NPM1*, *IDH2* (172), *KRAS* and *SMC3* for multivariate Cox analysis of intensively treated patients,
- gender, age, karyotype (normal vs abnormal), ELN risk (low/intermediate vs adverse), WHO status, *FLT3-ITD*, *KRAS*, *STAG2* and *TP53* for multivariate Cox analysis of palliatively treated patients.

Conditional inference tree analysis

Decision trees were formed by conditional inference tree (CIT) analyses whereby subpopulations were generated via recursive splitting of the studied population[3] using party version 1.3.3. The best predictor was searched for in each division resulting in 4 terminal nodes. Bonferroni correction was used due to the inclusion of multiple predicting factors. For CIT analyses of complete remission (CR) achievement we included i) all analyzed somatic genes as either “wild-type” or “mutated” and ii) differentially expressed genes (DEGs) from RNA-seq with P-values < 0.05 in univariate logistic regression analyses with regards to CR among the 20 most significant DEGs. No clinical variables were included since only gender, ELN risk and WHO status showed a significant impact on CR achievement (Supplementary Table S10 and Table S11). Gender was accounted for during the differential expression analysis (see below). ELN risk and WHO status were excluded since we aimed at developing a predictive algorithm based on leukemia-specific biology independent of ELN risk. Patients were assigned to either “low” or “high” groups based on median values of normalized count expressions for each gene. The final risk stratification model was based on the percentage of individuals who achieved CR within each final node.

Sample and library preparation, sequencing and preprocessing

Samples were separated for mononuclear cells and stored in isothermal liquid nitrogen freezers at -180 degrees Celsius. DNA and RNA was extracted using AllPrep DNA/RNA/Protein mini kit (Qiagen, Germany). DNA libraries were constructed using ThruPlex-FD (Rubicon Genomics), one aliquot was used for low- pass WGS and one aliquot was used for capture using the EZ SeqCap kit (Roche Nimblegen) as previously described[4]. The capture kit contained 655 genes known to be somatically mutated or associated with germline risk. Additionally, 242 pharmacogenic SNPs and 32 pharmacogenomic genes were also included[5].

Integrated transcriptomic and genomic analysis improves complete remission and survival predictions in elderly patients with acute myeloid leukemia

Collectively, the panel was 2.76 Mb in size. Sequencing was performed on Illumina HiSeq 2500. WGS libraries were sequenced to on average 0.5x coverage, captured libraries to 360X average coverage and RNA-seq libraries to a median of 33 million read-pairs per library (paired-end 2 x 101 bases). Preprocessing was performed using AutoSeq (https://github.com/dakl/autoseq), which includes best practices pipelines for the respective data types.

RNA was assessed using Bioanalyzer (Agilent, US) to ensure high quality (RNA Integrity Number > 8). One microgram of total RNA was used for rRNA depletion using RiboZero (Illumina, US) and stranded RNA-seq libraries were constructed using TruSeq Stranded Total RNA Library Prep Kit (Illumina, US). 2 x 100 paired end sequencing was performed on Illumina HiSeq 2500 (Illumina, US) at Science for Life Laboratory (Stockholm, Sweden).

Bioinformatic processing of RNA sequencing data

Insert sized ranged from approximately 50-300 bps. Standard Illumina adapters were trimmed using skewer version 0.1.117[6]. Alignment was carried out by STAR aligner version 2.4.0e[7] to the reference genome (GRCh37.73). PCR duplicates were marked but not removed, using Picard MarkDuplicates version 1.128 (http://broadinstitute.github.io/picard). Gene expression estimates were calculated with HTSeq count version 0.6.1[8]. DEseq2 version 1.22.1[9] was used for RNA-seq count data normalization and differential expression analysis. Following normalization, annotations where < 3 samples with normalized counts ≥ 5 were filtered out, resulting in 25769 genes used for downstream analysis. Differential expression analysis between patients who did or did not achieve CR was run using a multi-factor design, accounting for gender and *TP53* mutational status (~sex + *TP53* + CR). Analysis was conducted using R (https://www.R-project.org/) version 3.5.1, visualization of data was done with EhancedVolcano version 1.0.1.

Bioinformatic processing of DNA sequencing data

Standard Illumina adapters were trimmed using skewer version 0.1.117[6]. Alignment to GRCh37 in karyotypic order including decoy sequences and unplaced contigs was carried out using bwa mem version 0.7.7[10]. Calling of somatic SNVs was carried out with MuTect version 1.1.6[11] and small indels with pindel version 0.2.5a3[12], converted to VCF using the pindel2vcf tool in the pindel package and filtered using the custom SomaticPindelFilter walker version 0.1.2.

To enable detection of *FLT3-ITD* the ITD-mode of pindel was applied allowing for the detection of large insertions or deletions, using the parameters "-r false -k false -s false -t true -l false". Due to low panel coverage in exon 11 of *NPM1*, the RNA-seq data was used for detection of mutations, which has been robustly been applied previously[13]. Variants were annotated with snpEff version 4.0[14]. Silent mutations, and those in Introns, RNA, UTRs, Flanks, IGRs, and the ubiquitous Targeted_Region were excluded. Multiallelic variants were split using vcf_parser version 1.4 and normalized using bcftools norm version 1.2. As we did not have access to germline control DNA, 25 genes were manually curated for the identification of somatic variants relevant for prognostication. The ExAC database was used to filter out germline polymorphisms with allele frequency >0.01 in Caucasians[15]. For each individual, all variants with variant allele fraction <0.05 were removed to avoid false positive calls. Subsequently, each of the 25 genes was manually curated through an extensive literature search to identify variation of functional relevance. For each gene, only mutations harboring functional relevance was kept and used to stratify the patients.

Literature search for the 20 most significant genes from RNA-seq analysis

We searched the published literature on https://www.ncbi.nlm.nih.gov/pubmed for the 20 most significant genes from our RNA-seq analysis. The expression levels of these genes were included in the conditional inference tree analyses described below. The search terms used were (((gene AND leukemia) OR (gene AND cancer) AND (prognosis OR diagnosis) AND english[Language])) were the term 'gene' was altered accordingly. The search was performed in June 2019. Publications investigating primary tumor samples were included.

Supplementary Tables

Table S1. Laboratory values at time of diagnosis, median (range)				
	**All patients n = 182**	**Intensive treatment n = 130**	**Palliative treatment n = 45**	**P-value***
**Bone marrow blasts (%)**	57.0 (14.0 - 94.0)	54.5 (14.0 - 92.0)	70.0 (28.0 - 94.0)	0.03
Missing (n, %)	61 (33.5)	40 (30.8)	21 (46.7)	
**Peripheral blasts (x10⁹/L)**	5.3 (0.0 - 233.3)	6.1 (0.0 - 130.0)	8.7 (0.0 - 233.3)	0.33
Missing (n, %)	65 (35.7)	44 (33.9)	20 (44.4)	
**Hemoglobin (g/L)**	96.0 (45.0 - 148.0)	94.0 (45.0 - 148.0)	99.0 (70.0 - 119.0)	0.23
Missing (n, %)	59 (32.4)	40 (30.8)	19 (42.2)	
**Thrombocytes (x10⁹/L)**	67.0 (8.0 - 658.0)	70.0 (8.0 - 658.0)	56.5 (8.0 - 220.0)	0.05
Missing (n, %)	59 (32.4)	40 (30.8)	19 (42.2)	
**White blood cells (x10⁹/L)**	12.7 (0.5 - 301.5)	14.2 (0.5 - 153.6)	21.0 (0.5 - 301.5)	0.26
Missing (n, %)	59 (32.4)	40 (30.8)	19 (42.2)	
**Neutrophils (x10⁹/L)**	0.9 (0.0 - 37.0)	0.9 (0.0 - 37.0)	1.4 (0.0 - 13.0)	0.57
Missing (n, %)	66 (36.3)	44 (33.9)	21 (46.7)	
**Lactate dehydrogenase (µkat/L)**	5.0 (2.0 - 132.0)	5.3 (2.0 - 132.0)	5.1 (3.2 - 15.0)	0.95
Missing (n, %)	79 (43.4)	50 (38.5)	28 (62.2)	

Footnote: *P-values for comparisons between treatment groups using the Mann-Whitney U test.

Table S2. Mutation frequency, n (%).

Gene function	Gene	All patients n = 182	Patients with *de novo* AML n = 132	Intensive treatment n = 130	Palliative treatment n = 45	P-value*
**Nucleophosmin**	***NPM1***	58 (31.9)	49 (37.1)	44 (33.9)	13 (28.9)	0.58
**Chromatin remodeling**	***ASXL1***	29 (15.9)	17 (12.9)	18 (13.9)	8 (17.8)	0.63
	EZH2	6 (3.3)	6 (4.5)	4 (3.1)	2 (4.4)	0.65
**Transcription factors**	***CEBPA* (single)**	16 (8.8)	13 (9.8)	13 (10.0)	3 (6.7)	0.76
	***CEBPA* (double)**	5 (2.8)	3 (2.3)	5 (3.9)	0 (0.0)	0.33
	RUNX1	33 (18.1)	21 (15.9)	23 (17.7)	10 (22.2)	0.51
**Methylation-related**	***DNMT3A***	45 (24.7)	37 (28.0)	34 (26.2)	8 (17.8)	0.31
	IDH1	17 (9.3)	17 (12.9)	11 (8.5)	3 (6.7)	1.00
	***IDH2* (140)**	31 (17.0)	26 (19.7)	24 (18.5)	6 (13.3)	0.50
	***IDH2* (172)**	11 (6.0)	10 (7.6)	9 (6.9)	2 (4.4)	0.73
	TET2	51 (28.0)	32 (24.4)	37 (28.5)	12 (26.7)	1.00
**Kinases**	***FLT3* (D835)**	6 (3.3)	5 (3.8)	5 (3.9)	1 (2.2)	1.00
	FLT3-ITD	44 (24.2)	35 (26.5)	32 (24.6)	12 (26.7)	0.84
	KIT	2 (1.1)	2 (1.5)	2 (1.5)	0 (0.0)	1.00
**RAS pathway**	***PTPN11***	8 (4.4)	5 (3.8)	6 (4.6)	2 (4.4)	1.00
	KRAS	4 (2.2)	2 (1.5)	3 (2.3)	1 (2.2)	1.00
	NRAS	17 (9.3)	14 (10.6)	14 (10.8)	2 (4.4)	0.25
**Tumor suppressors**	***TP53***	25 (13.7)	17 (12.9)	16 (12.3)	8 (17.8)	0.45
	PHF6	5 (2.8)	3 (2.3)	4 (3.1)	1 (2.2)	1.00
	WT1	3 (1.7)	2 (1.5)	2 (1.5)	1 (2.2)	1.00
	PTEN	0 (0.0)	0 (0.0)	0 (0.0)	0 (0.0)	1.00
**Cohesin complex**	***RAD21***	7 (3.9)	5 (3.8)	4 (3.1)	3 (6.7)	0.38
	SMC1A	4 (2.2)	4 (3.0)	3 (2.3)	0 (0.0)	0.57
	SMC3	6 (3.3)	4 (3.0)	4 (3.1)	2 (4.4)	0.65
	STAG2	22 (12.1)	16 (12.1)	15 (11.5)	4 (8.9)	0.78
**Spliceosome**	***SF3B1***	3 (1.7)	3 (2.3)	2 (1.5)	1 (2.2)	1.00
	SRSF2	43 (23.6)	29 (22.0)	29 (22.3)	12 (26.7)	0.55
	U2AF1	13 (7.1)	6 (4.5)	7 (5.4)	5 (11.1)	0.19

Footnotes: *P-values for comparisons between treatment groups using Fisher's exact test.

Table S3. Univariate Cox regression for overall survival with regards to clinical data in intensively treated patients, n = 130.

Variable	Hazard ratio	95% confidence interval	P-value
**Gender (men vs women)**	0.60	0.40 - 0.90	0.01
**Age***	1.02	0.98 - 1.06	0.34
**Hemoglobin***	0.99	0.97 - 1.00	0.08
**Thrombocytes***	1.00	0.99 - 1.00	0.91
**White blood cells***	1.00	0.99 - 1.01	0.26
**Bone marrow blasts***	1.00	0.99 - 1.01	0.55
**Peripheral blasts***	1.00	0.99 - 1.01	0.26
**Lactate dehydrogenase***	1.07	1.02 - 1.11	0.002
**Neutrophils***	1.01	0.97 - 1.04	0.72
**WHO status****	1.68	1.35 - 2.08	< 0.001
**WHO status (0 vs 1)**	1.83	1.08 - 3.11	0.03
**WHO status (0 vs 2)**	3.40	1.71 - 6.75	< 0.001
**WHO status (0 vs 3)**	3.91	1.76 - 8.69	< 0.001
**WHO status (0 vs 4)**	14.22	3.15 - 64.15	< 0.001
**ELN risk*****	1.46	1.11 - 1.92	0.01
**ELN risk (Low vs Intermediate)**	1.49	0.82 - 2.70	0.19
**ELN risk (Low vs High)**	2.15	1.21 - 3.80	0.01
**ELN risk (Intermediate vs High)**	1.47	0.93 - 2.34	0.10
**AML etiology (*de novo* vs AHD-AML)**	1.74	0.98 - 3.09	0.06
**AML etiology (*de novo* vs t-AML)**	0.98	0.51 - 1.90	0.95
**Achieved complete remission (yes vs no)**	6.70	4.30 - 10.45	< 0.001

Abbreviations: WHO; World Health Organization, ELN; European LeukemiaNet, AHD-AML; AML with an antecedent hematological disorder, t-AML; therapy-related AML
Footnotes: *Continuous variables, i.e. HRs for every one-unit increase. **Continuous variable, i.e. HR for every one-unit increase in WHO status going from 0 to 4. ***Continuous variable, i.e. HR for every one-unit increase in ELN risk going from low to intermediate to high.

Table S4. Univariate Cox regression for overall survival in intensively treated patients ≥ 65 years, n = 130.

Gene function	Variable	Hazard ratio	95% CI	P-value
**Nucleophosmin**	***NPM1***	0.50	0.32 - 0.79	0.003
**Chromatin remodeling**	***ASXL1***	1.73	0.98 - 3.04	0.06
	EZH2	1.87	0.69 - 5.12	0.22
**Transcription factors**	***CEBPA* (single)**	0.70	0.35 - 1.39	0.31
	***CEBPA* (double)**	0.97	0.35 - 2.65	0.95
	RUNX1	0.99	0.56 - 1.74	0.96
**Methylation-related**	***DNMT3A***	1.02	0.64 - 1.61	0.93
	IDH1	0.97	0.45 - 2.11	0.94
	***IDH2* (140)**	0.74	0.43 - 1.27	0.27
	***IDH2* (172)**	0.50	0.22 - 1.14	0.09
	TET2	1.16	0.75 - 1.78	0.50
**Kinases**	***FLT3* (D835)**	0.48	0.15 - 1.51	0.21
	FLT3-ITD	1.02	0.64 - 1.62	0.93
	KIT	1.31	0.32 - 5.36	0.70
**RAS pathway**	***PTPN11***	0.53	0.17 - 1.66	0.27
	KRAS	2.48	0.78 - 7.87	0.12
	NRAS	1.06	0.53 - 2.11	0.87
**Tumor suppressors**	***TP53***	2.41	1.40 - 4.14	0.002
	PHF6	1.88	0.59 - 6.00	0.29
	WT1	0.37	0.05 - 2.63	0.32
	PTEN	—	—	—
**Cohesin complex**	***RAD21***	1.49	0.55 - 4.06	0.44
	SMC1A	0.66	0.09 - 4.75	0.68
	SMC3	0.31	0.08 - 1.27	0.10
	STAG2	0.90	0.48 - 1.69	0.75
**Spliceosome**	***SF3B1***	1.10	0.27 - 4.48	0.89
	SRSF2	0.83	0.50 - 1.37	0.46
	U2AF1	1.59	0.69 - 3.67	0.27

Table S5. Univariate Cox regression for relapse-free survival in intensively treated patients ≥ 65 years, n = 76*.

Gene function	Variable	Hazard ratio	95% CI	P-value
**Nucleophosmin**	***NPM1***	0.51	0.29 - 0.91	0.02
**Chromatin remodeling**	***ASXL1***	1.98	0.90 - 4.32	0.09
	EZH2	—	—	—
**Transcription factors**	***CEBPA* (single)**	0.89	0.38 - 2.10	0.79
	***CEBPA* (double)**	1.32	0.32 - 5.47	0.70
	RUNX1	0.64	0.27 - 1.51	0.31
**Methylation-related**	***DNMT3A***	0.88	0.46 - 1.69	0.70
	IDH1	1.03	0.32 - 3.33	0.96
	***IDH2* (140)**	0.61	0.31 - 1.23	0.17
	***IDH2* (172)**	0.87	0.37 - 2.03	0.74
	TET2	1.43	0.79 - 2.59	0.24
**Kinases**	***FLT3* (D835)**	0.70	0.21 - 2.26	0.55
	FLT3-ITD	1.09	0.60 - 1.98	0.79
	KIT	3.73	0.50 - 27.95	0.20
**RAS pathway**	***PTPN11***	0.95	0.30 - 3.07	0.94
	KRAS	—	—	—
	NRAS	0.44	0.11 - 1.80	0.25
**Tumor suppressors**	***TP53***	4.75	1.41 - 15.99	0.01
	PHF6	1.34	0.33 - 5.55	0.68
	WT1	0.42	0.06 - 3.10	0.40
	PTEN	—	—	—
**Cohesin complex**	***RAD21***	2.14	0.52 - 8.88	0.29
	SMC1A	—	—	—
	SMC3	0.65	0.16 - 2.69	0.55
	STAG2	1.11	0.50 - 2.49	0.80
**Spliceosome**	***SF3B1***	1.97	0.27 - 14.50	0.50
	SRSF2	0.93	0.48 - 1.81	0.82
	U2AF1	1.63	0.59 - 4.55	0.35

Footnotes: *77 intensively treated patients achieved complete remission (CR) but 1 lacked date of CR and thereby omitted.

Table S6. Univariate logistic regression analyses for mutational status in intensively treated patients with regards to death within 30 days from date of diagnosis, n = 130.

Gene function	Gene	Death within 30 days, n = 21	Survival > 30 days n = 109	Odds ratio	95% confidence interval	P-value*
**Nucleophosmin**	***NPM1***	7 (33.3)	37 (33.9)	1.028	0.391 - 2.913	0.96
**Chromatin remodeling**	***ASXL1***	3 (14.3)	15 (13.8)	0.957	0.279 - 4.432	0.95
	EZH2	1 (4.8)	3 (2.8)	0.566	0.068 - 11.756	0.63
**Transcription factors**	***CEBPA* (single)**	1 (4.8)	12 (11.0)	2.474	0.447 - 46.337	0.40
	***CEBPA* (double)**	0 (0.0)	5 (4.6)	—	—	1.00
	RUNX1	3 (14.3)	20 (18.3)	1.348	0.406 - 6.143	0.66
**Methylation-related**	***DNMT3A***	6 (28.6)	28 (25.7)	0.864	0.317 - 2.618	0.78
	IDH1	2 (9.5)	9 (8.3)	0.855	0.200 - 5.897	0.85
	***IDH2* (140)**	5 (23.8)	19 (17.4)	0.676	0.231 - 2.263	0.49
	***IDH2* (172)**	0 (0.0)	9 (8.3)	—	—	0.35
	TET2	6 (28.6)	31 (28.4)	0.994	0.367 - 3.000	0.99
**Kinases**	***FLT3* (D835)**	1 (4.8)	4 (3.7)	0.762	0.106 - 15.319	0.81
	FLT3-ITD	4 (19.0)	28 (25.7)	1.469	0.493 - 5.432	0.52
	KIT	0 (0.0)	2 (1.8)	—	—	1.00
**RAS pathway**	***PTPN11***	0 (0.0)	6 (5.5)	—	—	0.59
	KRAS	1 (4.8)	2 (1.8)	0.374	0.034 - 8.267	0.43
	NRAS	4 (19.0)	10 (9.2)	0.429	0.127 - 1.704	0.19
**Tumor suppressors**	***TP53***	5 (23.8)	11 (10.1)	0.359	0.114 - 1.261	0.09
	PHF6	1 (4.8)	3 (2.8)	0.566	0.068 - 11.756	0.63
	WT1	0 (0.0)	2 (1.8)	—	—	1.00
	PTEN	0 (0.0)	0 (0.0)	—	—	1.00
**Cohesin complex**	***RAD21***	1 (4.8)	3 (2.8)	0.566	0.068 - 11.756	0.63
	SMC1A	1 (4.8)	2 (1.8)	0.374	0.034 - 8.267	0.43
	SMC3	0 (0.0)	4 (3.7)	—	—	1.00
	STAG2	1 (4.8)	14 (12.8)	2.947	0.543 - 54.917	0.31
**Spliceosome**	***SF3B1***	0 (0.0)	2 (1.8)	—	—	1.00
	SRSF2	4 (19.0)	25 (22.9)	1.265	0.421 - 4.699	0.70
	U2AF1	0 (0.0)	7 (6.4)	—	—	0.60

Footnotes: *P-values for comparisons between groups using Fisher's exact test for genes with n = 0 in either group.

Table S7. Univariate Cox regression for overall survival with regards to mutational status in palliatively treated patients, n = 45.

Gene function	Variable	Hazard ratio	95% confidence interval	P-value*
**Nucleophosmin**	***NPM1***	1.12	0.56 - 2.24	0.75
**Chromatin remodeling**	***ASXL1***	0.99	0.45 - 2.15	0.97
	EZH2	1.30	0.31 - 5.47	0.72
**Transcription factors**	***CEBPA* (single)**	1.15	0.35 - 3.78	0.81
	***CEBPA* (double)**	—	—	—
	RUNX1	1.07	0.53 - 2.19	0.85
**Methylation-related**	***DNMT3A***	0.87	0.40 - 1.91	0.73
	IDH1	1.45	0.44 - 4.76	0.54
	***IDH2* (140)**	1.25	0.52 - 2.98	0.62
	***IDH2* (172)**	0.44	0.10 - 1.85	0.26
	TET2	0.95	0.47 - 1.93	0.90
**Kinases**	***FLT3* (D835)**	4.10	0.52 - 32.07	0.18
	FLT3-ITD	3.88	1.88 - 8.02	< 0.001
	KIT	—	—	—
**RAS pathway**	***PTPN11***	2.24	0.51 - 9.74	0.28
	KRAS	4.10	0.52 - 32.07	0.18
	NRAS	0.61	0.15 - 2.57	0.50
**Tumor suppressors**	***TP53***	1.80	0.80 - 4.05	0.16
	PHF6	1.90	0.25 - 14.24	0.53
	WT1	1.23	0.17 - 9.06	0.84
	PTEN	—	—	—
**Cohesin complex**	***RAD21***	1.37	0.42 - 4.53	0.60
	SMC1A	—	—	—
	SMC3	1.10	0.26 - 4.58	0.90
	STAG2	0.39	0.12 - 1.30	0.13
**Spliceosome**	***SF3B1***	2.30	0.30 - 17.35	0.42
	SRSF2	1.01	0.51 - 1.91	0.99
	U2AF1	1.20	0.47 - 3.08	0.71

Footnotes: *P-values for comparisons between groups using Fisher's exact test.

Table S8. Univariate Cox regression for overall survival with regards to clinical data in palliatively treated patients, n = 45.			
**Variable**	**Hazard ratio**	**95% confidence interval**	**P-value**
**Gender (male vs female)**	1.60	0.87 - 2.93	0.13
**Age***	1.04	0.98 - 1.10	0.17
**Hemoglobin***	0.99	0.96 - 1.03	0.93
**Thrombocytes***	1.001	0.99 - 1.01	0.86
**White blood cells***	1.001	1.00 - 1.01	0.77
**Bone marrow blasts***	1.021	1.00 - 1.04	0.07
**Peripheral blasts***	0.999	0.99 - 1.01	0.92
**Lactate dehydrogenase***	1.27	1.05 - 1.54	0.02
**Neutrophils***	1.11	0.96 - 1.29	0.15
**WHO status****	1.19	0.84 - 1.69	0.33
**Karyotype (normal vs abnormal)**	3.60	1.54 - 8.42	0.003
**ELN risk*****	2.20	1.16 - 8.42	0.003
**ELN risk (Low/Intermediate vs High)**	2.39	1.07 - 5.33	0.03
**AML etiology (*de novo* vs AHD-AML)**	0.91	0.45 - 1.84	0.79
**AML etiology (*de novo* vs t-AML)**	0.71	0.29 - 1.76	0.46

Abbreviations: WHO; World Health Organization, ELN; European LeukemiaNet, AHD-AML; AML with an antecedent hematological disorder, t-AML; therapy-related AML
Footnotes: *Continuous variables, i.e. HRs for every one-unit increase. **Continuous variable, i.e. HR for every one-unit increase in WHO status going from 0 to 4. ***Continuous variable, i.e. HR for every one-unit increase in ELN risk going from low to intermediate to high.

Table S9. Univariate logistic regression analyses for mutational status in intensively treated patients with regards to achieved complete remission (CR), n = 126.

Gene function	Gene	Achieved CR n = 77	Did not achieve CR n = 49	Odds ratio	95% confidence interval	P-value*
**Nucleophosmin**	***NPM1***	32 (41.6)	12 (24.5)	0.46	0.20 - 0.99	0.05
**Chromatin remodeling**	***ASXL1***	11 (14.3)	6 (12.2)	0.84	0.27 - 2.37	0.74
	EZH2	0 (0.0)	2 (4.1)	—	—	0.15
**Transcription factors**	***CEBPA*** **(single)**	10 (13.0)	3 (6.1)	0.44	0.09 - 1.52	0.23
	CEBPA **(double)**	3 (3.9)	1 (2.0)	0.51	0.03 - 4.15	0.57
	RUNX1	13 (16.9)	10 (20.4)	1.26	0.50 - 3.15	0.62
**Methylation-related**	***DNMT3A***	20 (26.0)	13 (26.5)	1.03	0.45 - 2.31	0.95
	IDH1	5 (6.5)	6 (12.2)	2.01	0.57 - 7.35	0.27
	IDH2 **(140)**	16 (20.8)	8 (16.3)	0.74	0.28 - 1.86	0.54
	IDH2 **(172)**	7 (9.1)	1 (2.0)	0.21	0.01 - 1.22	0.15
	TET2	23 (29.9)	13 (26.5)	0.85	0.37 - 1.87	0.69
**Kinases**	***FLT3*** **(D835)**	4 (5.2)	1 (2.0)	0.38	0.02 - 2.67	0.39
	FLT3-ITD	22 (28.6)	10 (20.4)	0.64	0.26 - 1.48	0.31
	KIT	1 (1.3)	1 (2.0)	1.58	0.06 - 40.67	0.31
**RAS pathway**	***PTPN11***	6 (7.8)	0 (0.0)	—	—	0.08
	KRAS	0 (0.0)	1 (2.0)	—	—	0.39
	NRAS	7 (9.1)	7 (14.3)	1.67	0.54 - 5.19	0.37
**Tumor suppressors**	***TP53***	3 (3.9)	12 (24.5)	8.00	2.37 - 36.71	0.002
	PHF6	3 (3.9)	1 (2.0)	0.51	0.03 - 4.15	0.57
	WT1	2 (2.6)	0 (0.0)	—	—	0.52
	PTEN	0 (0.0)	0 (0.0)	—	—	1.00
**Cohesin complex**	***RAD21***	2 (2.6)	2 (4.1)	1.60	0.19 - 13.67	0.65
	SMC1A	1 (1.3)	2 (4.1)	3.23	0.30 - 70.72	0.34
	SMC3	4 (5.2)	0 (0.0)	—	—	0.16
	STAG2	10 (13.0)	5 (10.2)	0.76	0.22 - 2.30	0.64
**Spliceosome**	***SF3B1***	1 (1.3)	1 (2.0)	1.58	0.06 - 40.67	0.75
	SRSF2	18 (23.4)	11 (22.4)	0.95	0.39 - 2.21	0.90
	U2AF1	5 (6.5)	2 (4.1)	0.61	0.09 - 2.97	0.57

Abbreviations: CR; complete remission.
Footnotes: *P-values for comparisons between CR groups using Fisher's exact test for genes with n = 0 in either CR group.

Table S10. Univariate logistic regression analyses for clinical data in intensively treated patients with regards to achieved complete remission, n = 126.

Variable	Odds ratio	95% confidence interval	P-value
**Gender (male vs female)**	0.32	0.15 - 0.67	0.003
**Age***	1.04	0.97 - 1.12	0.26
**Hemoglobin***	0.99	0.97 - 1.02	0.49
**Thrombocytes***	1.00	1.00 - 1.00	0.95
**White blood cells***	1.00	0.99 - 1.02	0.46
**Bone marrow blasts***	1.01	0.99 - 1.03	0.26
**Peripheral blasts***	1.01	0.99 - 1.02	0.43
**Lactate dehydrogenase***	1.04	1.00 - 1.12	0.23
**Neutrophils***	1.02	0.95 - 1.10	0.53
**WHO status****	2.29	1.47 - 3.78	< 0.001
**Karyotype (normal vs abnormal)**	2.08	0.99 - 4.48	0.06
**ELN risk*****	1.86	1.12 - 3.15	0.02
**ELN risk (Low/Intermediate vs High)**	2.90	1.38 - 6.25	0.006
**AML etiology (*de novo* vs AHD-AML)**	1.35	0.44 - 4.04	0.59
**AML etiology (*de novo* vs t-AML)**	0.51	0.11 - 1.84	0.34

Abbreviations: WHO; World Health Organization, ELN; European LeukemiaNet, AHD-AML; AML with an antecedent hematological disorder, t-AML; therapy-related AML
Footnotes: *Continuous variables, i.e. HRs for every one-unit increase. **Continuous variable, i.e. HR for every one-unit increase in WHO status going from 0 to 4. ***Continuous variable, i.e. HR for every one-unit increase in ELN risk going from low to intermediate to high.

Table S11. Laboratory values at time of diagnosis of intensively treated patients with regards to achieved complete remission or not, n = 126, median (range).

Laboratory variable	Achieved CR n = 77	Did not achieve CR n = 49	P-value*
**Bone marrow blasts (%)**	50.0 (14.0 - 92.0)	58.5 (20.0 - 90.0)	0.25
Missing (n, %)	18 (23.4)	22 (44.9)	
**Peripheral blasts (x10^9/L)**	5.3 (0.0 - 130.0)	7.3 (0.0 - 121.3)	0.60
Missing (n, %)	22 (28.6)	22 (44.9)	
**Hemoglobin (g/L)**	95.5 (53.0 - 139.0)	93.5 (45.0 - 148.0)	0.43
Missing (n, %)	19 (24.7)	21 (42.9)	
**Thrombocytes (x10^9/L)**	74.0 (20.0 - 658.0)	58.0 (8.0 - 469.0)	0.50
Missing (n, %)	19 (24.7)	21 (42.9)	
**White blood cells (x10^9/L)**	14.2 (0.5 - 149.0)	21.6 (0.9 - 153.6)	0.64
Missing (n, %)	19 (24.7)	21 (42.9)	
**Neutrophils (x10^9/L)**	0.9 (0.0 - 35.6)	0.7 (0.0 - 23.8)	0.97
Missing (n, %)	23 (29.9)	21 (42.9)	
**Lactate dehydrogenase (µkat/L)**	5.7 (2.5 - 27.4)	5.0 (2.0 - 132.0)	0.59
Missing (n, %)	24 (31.2)	26 (53.1)	

Abbreviations: CR; complete remission
Footnotes: *P-values for comparisons between CR groups using the Mann-Whitney U test.

Table S12. The 20 most significant genes from RNA-seq analysis.

Gene	Log_2 fold change (no CR vs CR)	Adjusted P-value*	Previous cancer association with or without prognostic impact (PMID)°
ADAMTS2	2.32	0.006	colorectal cancer (30679934)<br>oral cancer (28176846, 16164832)
AVPR1A	2.78	0.007	breast cancer (27858316)
BAG3	-1.85	0.006	ALL (14614315)<br>pancreatic cancer (22944597, 31119886)<br>breast cancer (29644001)<br>chondrosarcoma (29484408)<br>eyelid carcinoma (28373462)<br>endometrial cancer (27414463)<br>medulloblastoma (27456361)<br>colorectal cancer (23553442, 26577854)<br>ovarian cancer (27120977, 24992675)<br>uterine cancer (25738313)<br>lung cancer (25149536, 21971866)<br>CLL (23978946)<br>HCC (24130164)<br>glioblastoma (21561597)
BCL2L10	2.26	0.002	MDS/AML (28514758, 22577154)<br>breast cancer (29330143, 22207111)<br>gastric cancer (20428828, 21166696)
DNAH2	4.88	< 0.001	CMML (26648538)
EEF1A2	-1.84	0.006	ovarian cancer (26059197, 18164751)<br>breast cancer (25971350, 16156888)<br>pancreatic cancer (23165190, 19138673)<br>prostate cancer (28923030, 22095224)<br>NSCLC (24510995)<br>gastric cancer (25601347)<br>cholangiocarcinoma (27082702)<br>colorectal cancer (30132996)
EEPD1	-0.99	0.003	-
FCER1A	1.72	0.01	-
FOXF2	5.82	< 0.001	breast cancer (23620774, 27377963)<br>HCC (25824262)<br>esophageal cancer (26137292, 28222662)<br>NSCLC (27487137)
HSPA6	-2.24	0.007	HCC (25798051)
ITLN1	-2.56	< 0.001	ovarian cancer (30988639)<br>colorectal cancer (29659199, 28422056)<br>prostate cancer (24659266)<br>gastric cancer (22083213, 25965823)<br>renal cancer (26539805)<br>mesothelioma (20628387)

Table S12. The 20 most significant genes from RNA-seq analysis.			
**Gene**	**Log_2 fold change (no CR vs CR)**	**Adjusted P-value***	**Previous cancer association with or without prognostic impact (PMID)°**
MET	-2.03	0.01	CLL (28231754)<br>multiple myeloma (25810013)<br>AML (22683780)<br>CML (18609087)<br>adult T-cell leukemia (12538467)<br>HCC (31186768)<br>colorectal (30556647, 31116909)<br>gastric cancer (29422968, 31056743)<br>head and neck cancer (30708403)<br>esophageal cancer (30610559)<br>lung cancer (29631966, 30583073)<br>breast cancer (30444219)<br>pancreatic cancer (29229329, 30343272)<br>cervical cancer (29058790)
MIR3135A	-1.51	0.005	-
MYRIP	2.04	0.002	pancreatic cancer (26345976)
NKAIN2	3.28	0.002	prostate cancer (27588475)
PLIN2	-1.40	0.006	renal cancer (29749470, 28975715)<br>malignant melanoma (27886404)<br>lung adenocarcinoma (27467545)<br>Burkitt lymphoma (22952953)<br>colorectal cancer (21828233)
RP11-883A18:3	-3.15	0.007	-
SIGLEC1	2.31	0.004	esophageal cancer (30516869)<br>bladder cancer (29520898)<br>breast cancer (30991022)<br>colorectal cancer (23734742)<br>malignant melanoma (26297710)<br>endometrial cancer (26991548)<br>Kaposi's sarcoma (12697073)
TNFSF14	-1.85	0.001	colon cancer (28249900)

Table S12. The 20 most significant genes from RNA-seq analysis.

Gene	Log_2 fold change (no CR vs CR)	Adjusted P-value*	Previous cancer association with or without prognostic impact (PMID)°
ZBTB7A	-0.66	0.007	AML (27252013, 26968532, 30251205)<br>prostate cancer (21251909)<br>gastric cancer (26404754)<br>malignant melanoma (25995384)<br>colorectal cancer (25367850, 31186640)<br>breast cancer (30265334, 21392388)<br>NSCLC (17907153, 28088736, 26451776)<br>HCC (21985851, 26164003)<br>oral cancer (25439053)<br>HL, FL, DLBCL (24326827)<br>chondrosarcoma (22847180)<br>ovarian cancer (21176152)

Abbreviations: CR; complete remission, ALL; acute lymphoblastic leukemia, AML; acute myeloid leukemia, MDS; myelodysplastic syndrome, HCC; hepatocellular carcinoma, CMML; chronic myelomonocytic leukemia, CLL; chronic lymphocytic leukemia, NSCLC; non-small cell lung cancer, DLBCL; diffuse large B-cell lymphoma, HL; Hodgkin's lymphoma, FL; follicular lymphoma.
Footnotes: *Adjusted by the Benjamini-Hochberg procedure. °Literature search as defined in Supplementary Methods.

Supplementary Figures

Legends

Figure S1. Flow chart. Flow chart of included/excluded patients.

Figure S2. Oncoplots by treatment regimen. Panel A-B; Oncoplot for intensively (n = 130, Panel A) and palliatively (n = 45, Panel B) treated patients. Each column corresponds to one patient, rows correspond to somatic mutations in included genes with colors related to AML etiology,

Figure S3. Overall survival by treatment regimen and *TP53* status. Kaplan-Meier estimates with log rank P = 0.04 when comparing *TP53*-mutated (*TP53*+) cases and P < 0.001 comparing patients with wild-type *TP53* (*TP53*-).

Figure S4. Overall survival in palliative patients by *FLT3-ITD* status.

Figure S5. Overall survival and CR. Kaplan-Meier estimates related to achievement of complete remission (CR) in intensively treated patients.

Figure S6. Rates of complete remission per risk group. Obtained rates of complete remission in original cohort and the beatAML cohort used for external validation. Abbreviations: CR; complete remission, Int; Intermediate.

Figure S1

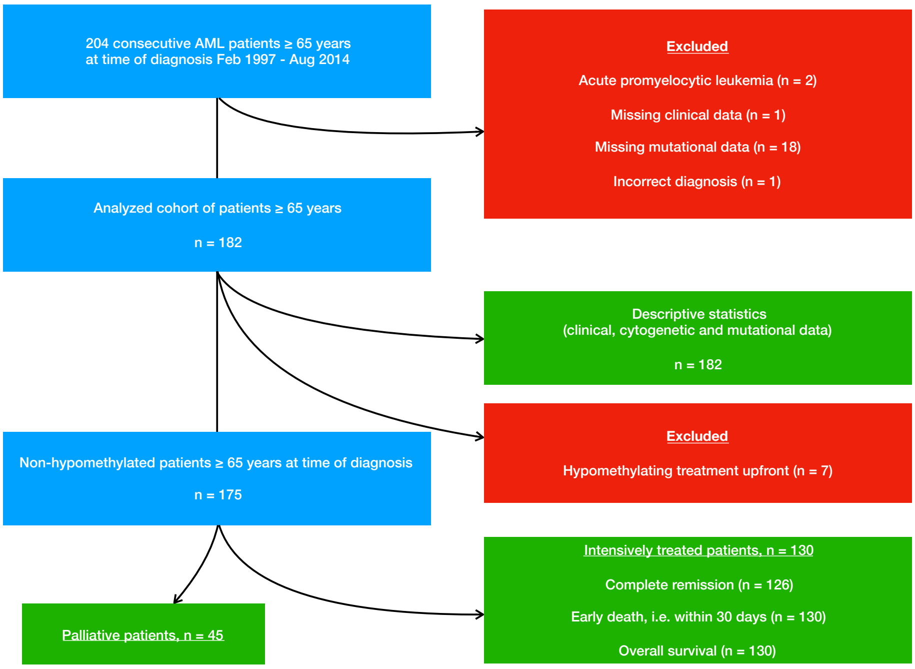
204 consecutive AML patients ≥ 65 years
at time of diagnosis Feb 1997 - Aug 2014
Excluded
Acute promyelocytic leukemia (n = 2)
Missing clinical data (n = 1)
Missing mutational data (n = 18)
Incorrect diagnosis (n = 1)
Analyzed cohort of patients ≥ 65 years
n = 182
Descriptive statistics
(clinical, cytogenetic and mutational data)
n = 182
Excluded
Hypomethylating treatment upfront (n = 7)
Non-hypomethylated patients ≥ 65 years at time of diagnosis
n = 175
Intensively treated patients, n = 130
Complete remission (n = 126)
Early death, i.e. within 30 days (n = 130)
Overall survival (n = 130)
Palliative patients, n = 45

Supplementary
Integrated transcriptomic and genomic analysis improves complete remission and survival predictions in elderly patients with acute myeloid leukemia

Figure S2, Panel A

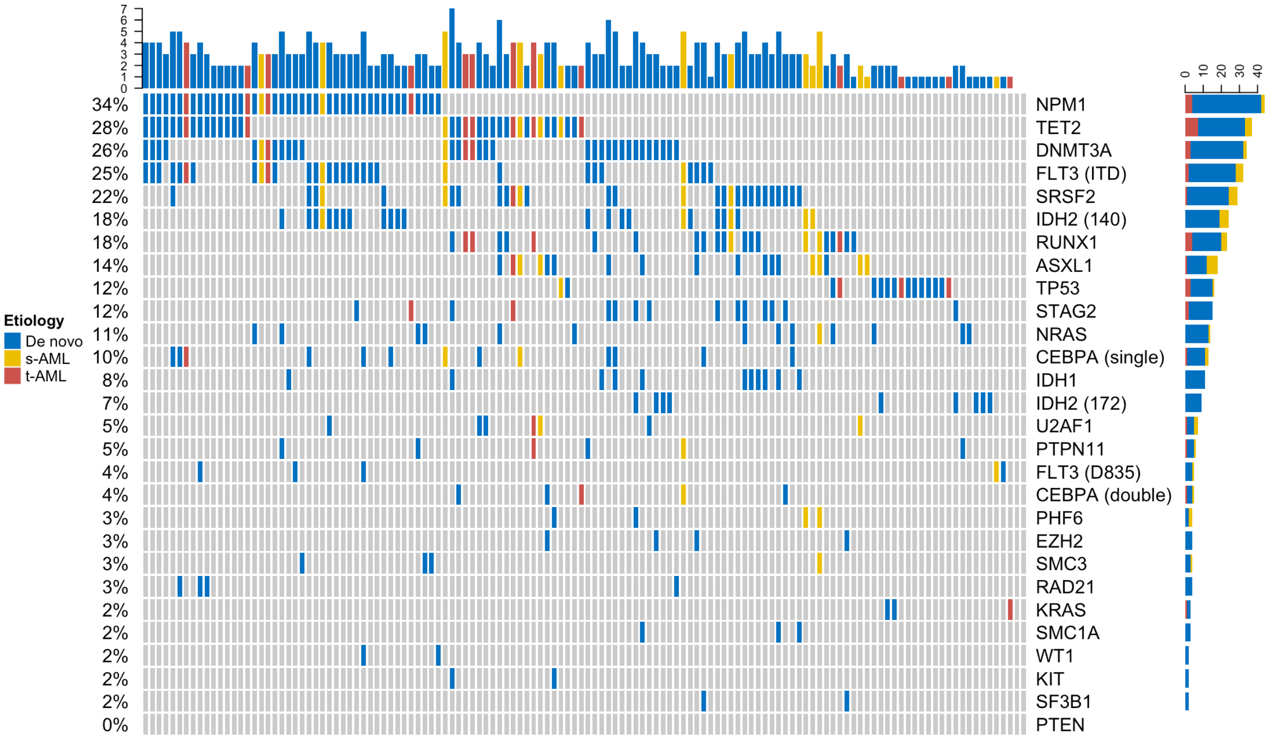

Figure S2, Panel B

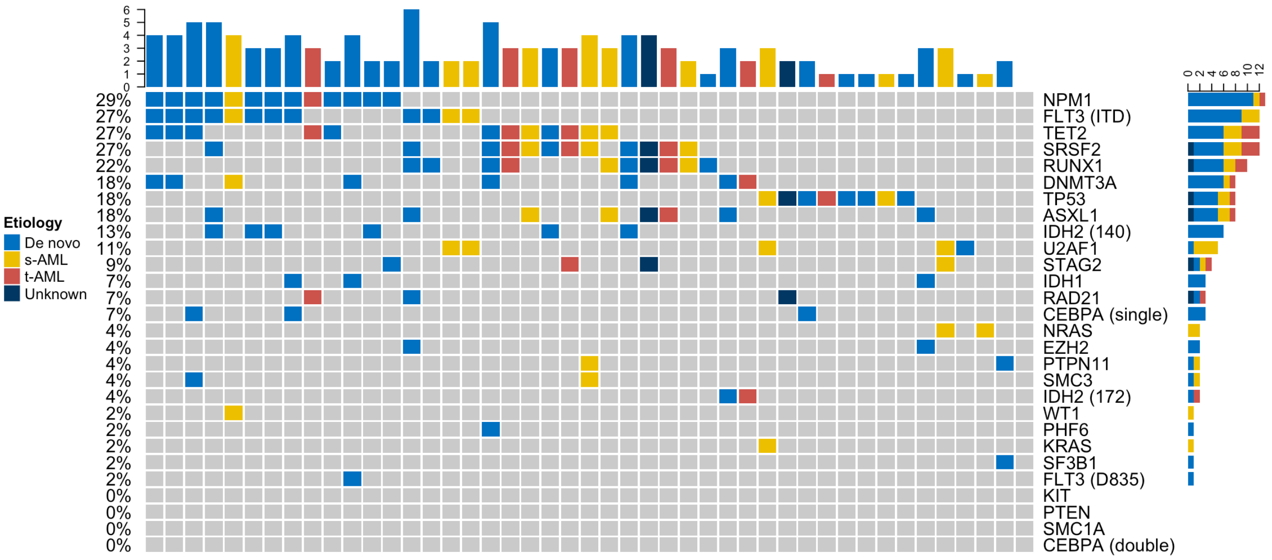

<u>Supplementary</u>

Integrated transcriptomic and genomic analysis improves complete remission and survival predictions in elderly patients with acute myeloid leukemia

Figure S3

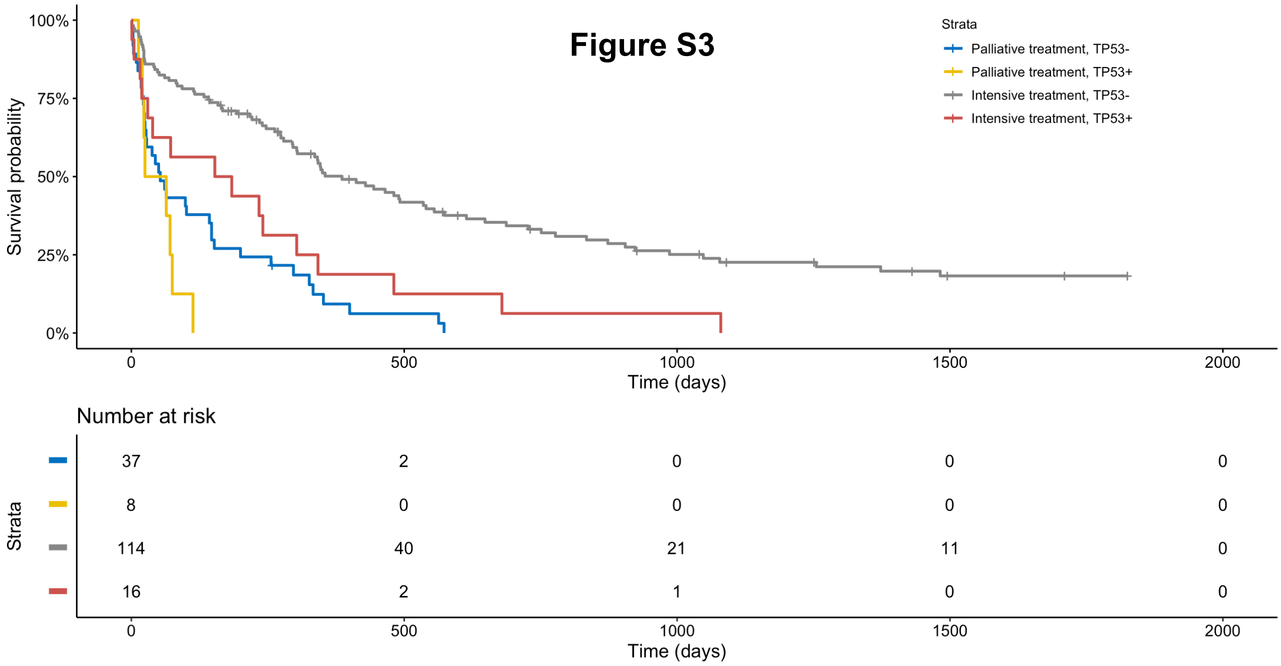

Figure S4

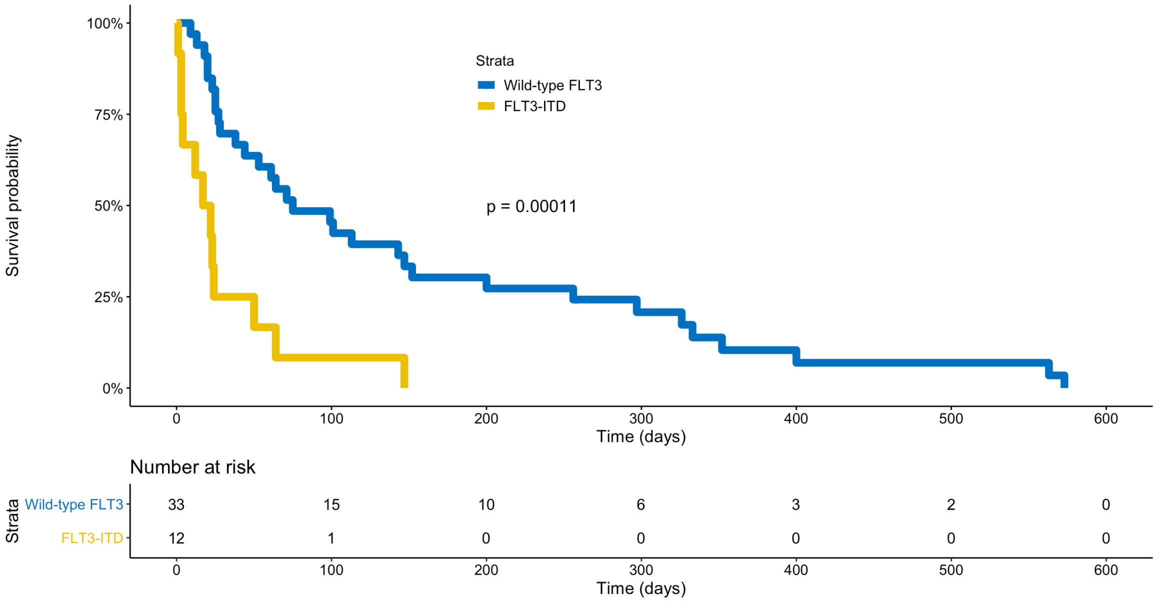

Figure S5

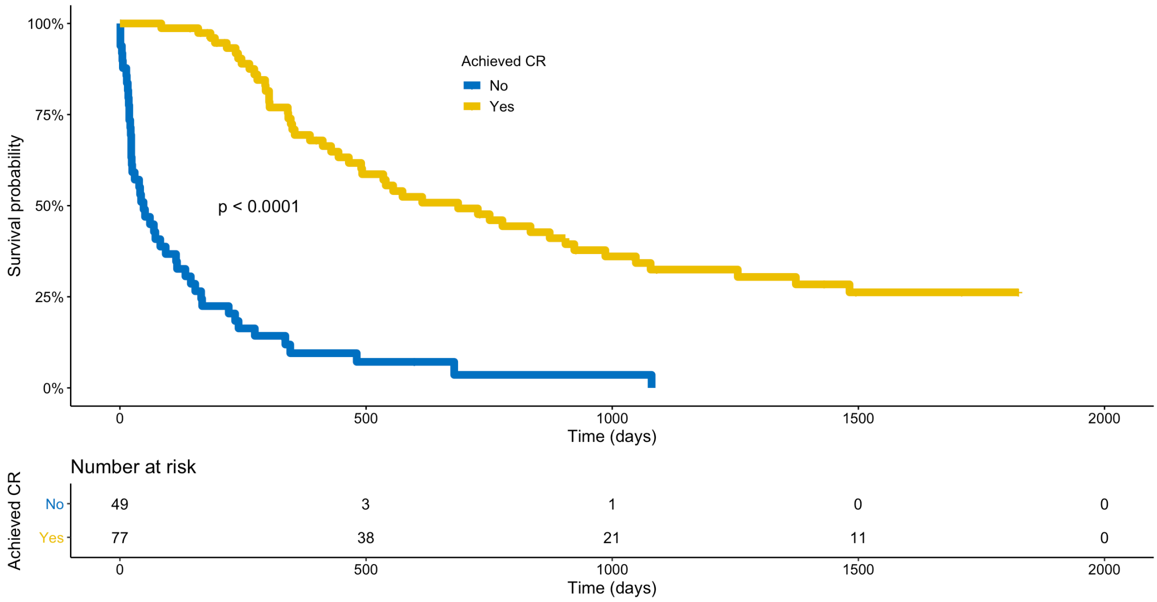

Figure S6

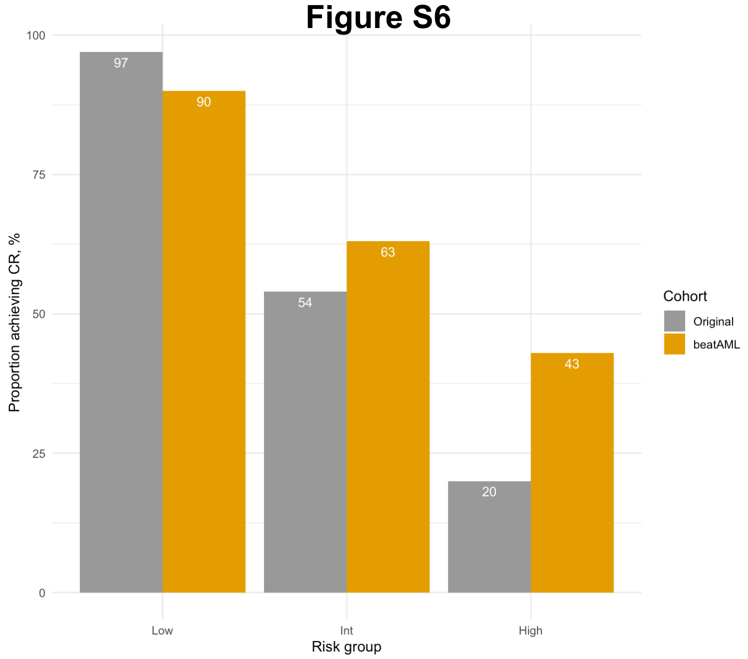

Supplementary References

1. Döhner H et al. Diagnosis and management of AML in adults: 2017 ELN recommendations from an international expert panel. *Blood.* 2017;129(4):424-447.

2. R Core Team (2017). R: A language and environment for statistical computing. R Foundation for Statistical Computing, Vienna, Austria. https://www.R-project.org/.

3. Hothorn T, Hornik K, Zeileis A. Unbiased Recursive Partitioning: A Conditional Inference Framework. *Journal of Computational and Graphical Statistics*. 2006;15(3):651–674.

4. Lindberg J et al. Exome sequencing of prostate cancer supports the hypothesis of independent tumour origins. *European urology*. 2013;63(2):347-53.

5. Wagle N et al. High- throughput detection of actionable genomic alterations in clinical tumor samples by targeted, massively parallel sequencing. *Cancer discovery*. 2012;2(1):82-93.

6. Jiang H, Lei R, Ding SW, Zhu S. Skewer: a fast and accurate adapter trimmer for next-generation sequencing paired-end reads. *BMC Bioinformatics*. 2014;15:182.

7. Dobin A et al. STAR: ultrafast universal RNA-seq aligner. *Bioinformatics*. 2013;29(1):15-21.

8. Anders S, Pyl PT, Huber W. HTSeq--a Python framework to work with high- throughput sequencing data. *Bioinformatics*. 2015;31(2):166-9.

9. Love MI, Huber W, Anders S. Moderated estimation of fold change and dispersion for RNA-seq data with DESeq2. *Genome Biol*. 2014;15(12):550.

10. Li H, Durbin R. Fast and accurate short read alignment with Burrows-Wheeler transform. *Bioinformatics*. 2009;25(14):1754-60.

11. Cibulskis K et al. Sensitive detection of somatic point mutations in impure and heterogeneous cancer samples. *Nat Biotechnol*. 2013;31(3):213-9.

12. Ye K, Schulz MH, Long Q, Apweiler R, Ning Z. Pindel: a pattern growth approach to detect break points of large deletions and medium sized insertions from paired-end short reads. *Bioinformatics*. 2009;25(21):2865-71.

13. Falini B et al. Cytoplasmic nucleophosmin in acute myelogenous leukemia with a normal karyotype. *N Engl J Med*. 2005;352(3):254-66.

14. Cingolani P et al. A program for annotating and predicting the effects of single nucleotide polymorphisms, SnpEff: SNPs in the genome of Drosophila melanogaster strain w1118; iso-2; iso-3. Fly (Austin). 2012;6(2):80-92.

15. Lek M et al. Analysis of protein-coding genetic variation in 60,706 humans. *Nature*. 2016;536(7616):285-91.
